# Development and validation of immune-based biomarkers and deep learning models for Alzheimer’s disease

**DOI:** 10.3389/fgene.2022.968598

**Published:** 2022-08-22

**Authors:** Yijie He, Lin Cong, Qinfei He, Nianping Feng, Yun Wu

**Affiliations:** Department of Neurology, The Second Affiliated Hospital of Harbin Medical University, Harbin, China

**Keywords:** Alzheimer’s disease, immune infiltration, biomarkers, random forest, artificial neural networks

## Abstract

**Background:** Alzheimer’s disease (AD) is the most common form of dementia in old age and poses a severe threat to the health and life of the elderly. However, traditional diagnostic methods and the ATN diagnostic framework have limitations in clinical practice. Developing novel biomarkers and diagnostic models is necessary to complement existing diagnostic procedures.

**Methods:** The AD expression profile dataset GSE63060 was downloaded from the NCBI GEO public database for preprocessing. AD-related differentially expressed genes were screened using a weighted co-expression network and differential expression analysis, and functional enrichment analysis was performed. Subsequently, we screened hub genes by random forest, analyzed the correlation between hub genes and immune cells using ssGSEA, and finally built an AD diagnostic model using an artificial neural network and validated it.

**Results:** Based on the random forest algorithm, we screened a total of seven hub genes from AD-related DEGs, based on which we confirmed that hub genes play an essential role in the immune microenvironment and successfully established a novel diagnostic model for AD using artificial neural networks, and validated its effectiveness in the publicly available datasets GSE63060 and GSE97760.

**Conclusion:** Our study establishes a reliable model for screening and diagnosing AD that provides a theoretical basis for adding diagnostic biomarkers for the AD gene.

## Introduction

Relevant studies have shown that in 2018 the prevalence of dementia is about 50 million people worldwide and is expected to triple by 2050 (Scheltens et al., 2021). Alzheimer’s disease (AD), the most common form of dementia, is a significant threat to the health and lives of older adults, with initial symptoms of memory loss, decreased verbal skills, and impaired logical thinking (Sabayan and Sorond, 2017). The onset of AD is insidious, and some pathophysiological changes are thought to occur years or even decades before the clinical diagnosis of dementia (Morris, 2005). It was not until 2011 that the concept of the preclinical stage of AD was explicitly introduced in the NIA-AA diagnostic criteria for Alzheimer’s disease (Sperling et al., 2011). The introduction of this concept is critical and suggests that interventions can be made in the preclinical stage of AD to ultimately delay the disease’s progression.

The latest NIA-AA AD diagnostic framework-ATN framework (Jack et al., 2018), officially published in 2018, is considered promising for the early identification of disease development in the preclinical phase of AD. In this framework, the diagnosis of AD is determined by the biomarkers Aβ and tau. Still, the framework is currently only used for scientific research and is not widely used in the clinic. Therefore, how identifying and diagnosing early becomes an urgent problem for us.

Genetic factors are considered a significant risk for Alzheimer’s disease, accounting for 60%–80% of the disease (Gatz et al., 2006). In addition to the well-known APEε4 risk alleles, there are many genes involved in AD that we do not recognize (Jansen et al., 2019). Second-generation sequencing technology has revealed the potential of some of these genes in the development of AD, such as SORL1 (Holstege et al., 2017), ABC47 (Bossaerts et al., 2021), TREM2, and R47H (Cheng-Hathaway et al., 2018; Sudom et al., 2018). With the development of science and technology, bioinformatics analysis has been widely used in diseases. Weighted co-expression networks (WGCNA) have become the most prevalent gene screening tool. They have been validated in numerous conditions by constructing free-scale gene co-expression networks to explore the association between clinical features and genes with co-expression patterns. In addition, some machine learning algorithms have been gradually introduced into medical research. Random Forest (RF) algorithms have been applied in acute myeloid leukemia (Shi and Xu, 2019), ALS (Hothorn and Jung, 2014), and cardiovascular diseases (Yang et al., 2020). Artificial neural networks have also demonstrated their powerful functions in medical research applications. Some scholars have validated diagnostic models for ulcerative colitis and heart failure (Li et al., 2020; Tian et al., 2020). Using WGCNA combined with machine learning to analyze AD biological data to find AD susceptibility genes may be a breakthrough.

**FIGURE 1:**
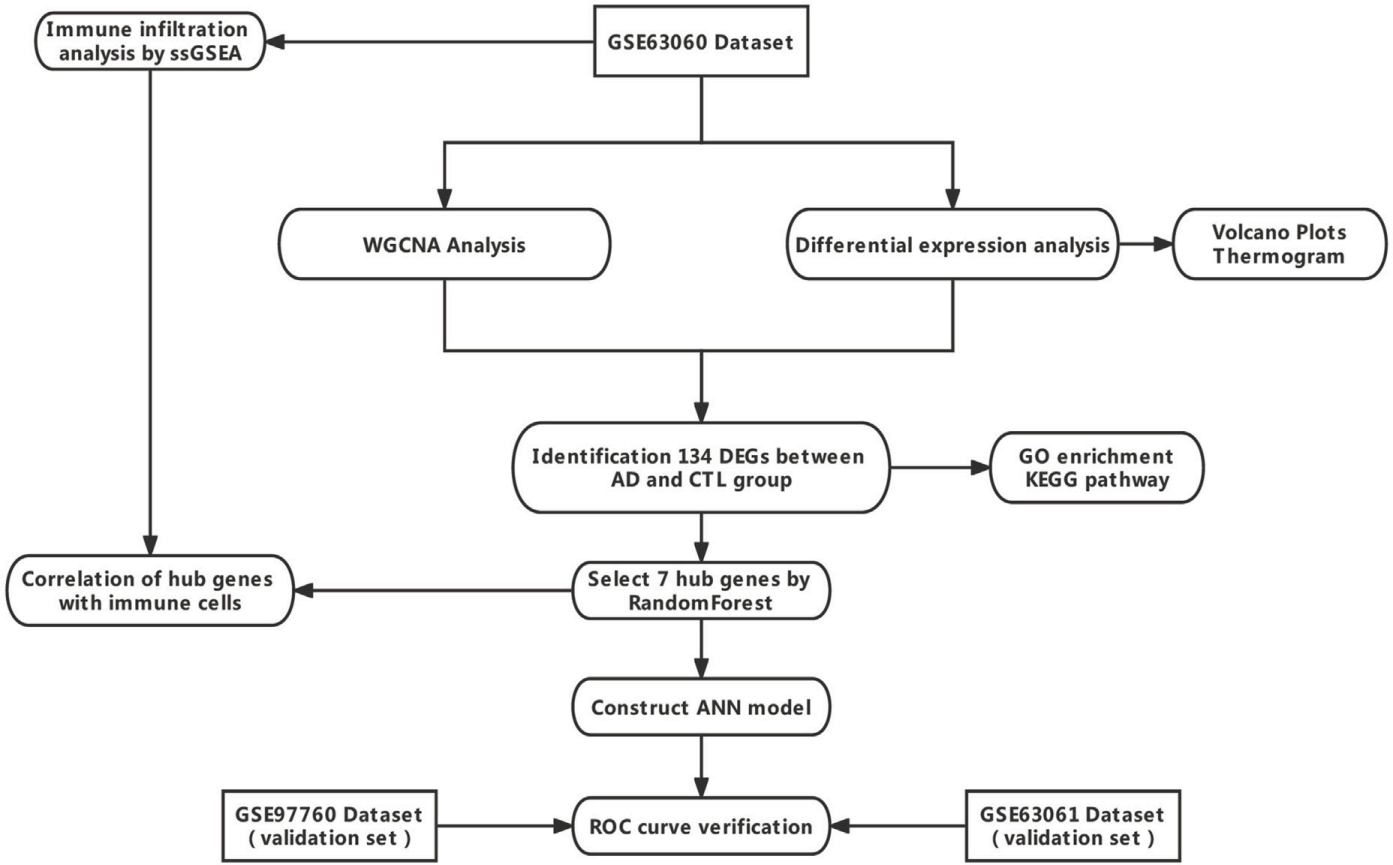
Technology route. Abbreviations: AD, Alzheimer’s Disease; CTL, Healthy Control; WGCNA, Weighted Gene Co-expression Network Analysis; ANN, Artificial Neural Network.

In this study, we identified datasets of AD serological sources in the GEO database, used WGCNA with differential expression analysis to screen out differentially expressed genes (DEGs) between AD and normal control samples from them, and applied the random forest algorithm to screen out hub genes, constructed an artificial neural network AD diagnostic model, and further analyzed the role of hub genes in the immune microenvironment to provide early identification and intervention of AD and a better understanding of the molecular immune mechanisms provide new perspectives. The technical route is shown in Figure 1.

## Materials and methods

### Download and processing of expression spectrum data

In this study, the AD gene expression profile datasets GSE63060, GSE63061, and GSE97760 were downloaded from Gene Expression Omnibus (Table 1). The annotation information of the microarray probes was obtained through the soft annotation list of the corresponding platform. Multiple probes corresponding to the same gene symbol may be encountered during the annotation of probe data. We use the average probe expression as the gene expression level. The process is annotated through a Perl language script (https://www.perl.org/).

**TABLE 1:** Dataset information from the GEO database.

Location	Dataset ID	Platform	Type	Number
Blood	GSE63060	GPL6947	Microarray	104 control vs. 145 AD
Blood	GSE63061	GPL10558	Microarray	134 control vs. 139 AD
Blood	GSE97760	GPL16699	Microarray	10 control vs. 9 AD

### Principal component analysis

**FIGURE 2:**
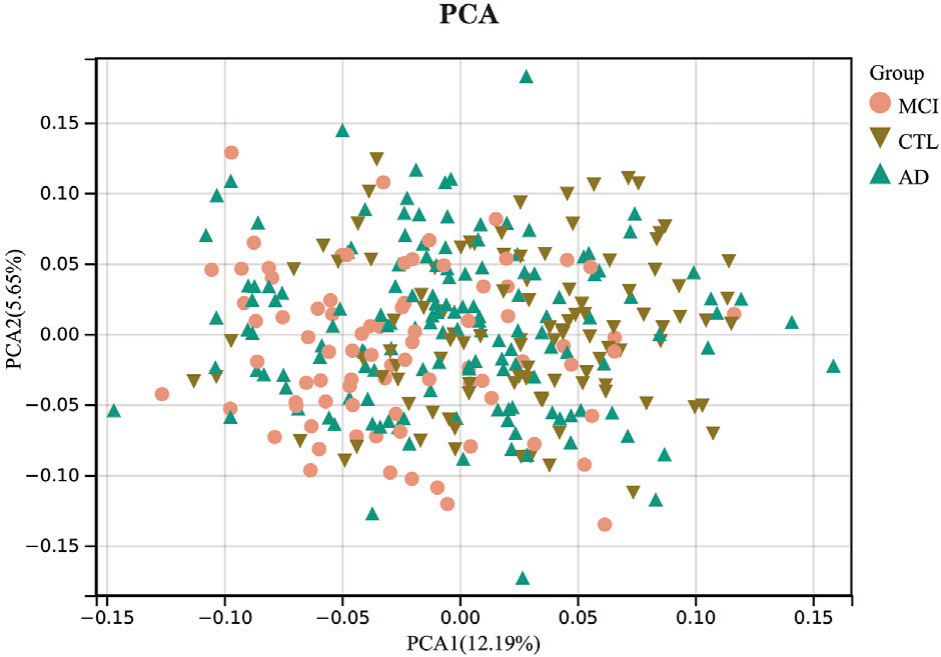
Principal component analysis.

To analyze the distribution of samples in the expression profiling dataset GSE63060 and the differences in gene expression between samples. We first performed a z-score on the expression spectrum, further performed dimensionality reduction analysis using the prcomp function to obtain the reduced matrix, and visualized the results. The results of PCA (Figure 2) show that there is little variability between the AD and CTL groups, so constructing a diagnostic model is necessary. We have invoked the R package stats (version 3.6.0) for the above procedure.

### Differential expression analysis

We performed differential expression analysis on the expression profiling dataset GSE63060 for differential expression genes between the AD and CTL groups with the R package limma (version 3.40.6) (Ritchie et al., 2015). The significance criteria for DEGs were set to FoldChang >1.2 and adjusted *p*-value < 0.05. Heatmaps of DEGs were implemented by the pheatmap package (version 1.0.12), and differentially expressed genes were represented by volcano maps constructed by the ggplot2 package (version 3.3.5).

### Construction of weighted gene co-expression network analysis

First, the obtained expression profile matrix was read in. The MAD value, also known as median absolute deviation, was calculated separately for each gene. The first 50% of genes with the smallest MAD values were eliminated. The goodSampleGenes function of the R package WGCNA was used to eliminate outlier genes and samples, on which the scale-free co-expression network was further constructed. The genes were then hierarchically clustered to identify modules. Pearson’s correlation analysis determined correlations between clinical phenotypes and the resulting modules. Among all the obtained modules, we selected the most correlated modules with the normal control group (CTL group) and AD group for further analysis. The genes in the key modules were those that met the following criteria: gene significance (GS) > 0.1 and module membership (MM) > 0.8.

### Overlapping weighted co-expression networks-related module genes with differential expression genes

The 200 Differential Expression genes obtained from the differential expression analysis overlapped with the WGCNA correlation module genes. Venn (Bardou et al., 2014) diagrams were used to visualize the results.

### Screening for hub genes using random forest

Random forest models for differentially expressed genes were constructed using the randomForest package (version 4.6–14). First, the number of decision trees needed to achieve the highest model accuracy in cross-validation was calculated based on the expression matrix of differentially expressed genes. Second, the random forest model was constructed. The importance value scores of dimensions were obtained from the random forest model using the Gini coefficient method. Genes with an importance value greater than two were identified as hub genes for subsequent analysis. The hub genes were clustered, and heatmaps were drawn with the pheatmap package (version 1.0.12).

### Correlation analysis between hub genes and immune characteristics

To determine the role of hub genes in the immune microenvironment, we analyzed the correlation between them and immune cell infiltration by applying the ssGSEA approach to analyze the proportion of 28 different immune cell distributions and infiltration scores in each sample of the dataset GSE63060. Pheatmap package (version 1.0.12) was used to map the immune cell distribution maps. Further, the voplot package (version 0.3.7) was used to present the differences in immune cell infiltration scores between the CTL and AD groups. Finally, we used the Spearman correlation test to assess the correlation between hub genes and immune cells and visualized the results using the ggplot2 package (version 3.3.5).

### Construction and validation of artificial neural network models

The hub genes expression matrix obtained was extracted, and the data were first scaled by Min-Max processing. An artificial neural network model was constructed using the R package Neuralnet (version 1.44.2). The parameters were set to five hidden layers, the neural network algorithm obtained the gene weight information, and the disease classification score Neural AD was obtained using “Gene Expression” × “Gene_Weight.” The model was visualized using the software package NeuralNetTools (version 1.5.2). Finally, the classification performance of the artificial neural network model was evaluated using the pROC package (version 1.18.0) and the ggplot2 package (version 3.3.5) to calculate the AUC scores and plot the ROC curves. Two independent datasets, GSE63060 and GSE97760, were used to validate the accuracy of the artificial neural network model, while ROC curves were plotted and the area under the curve AUC was calculated.

## Results

### Differential expression analysis

**FIGURE 3:**
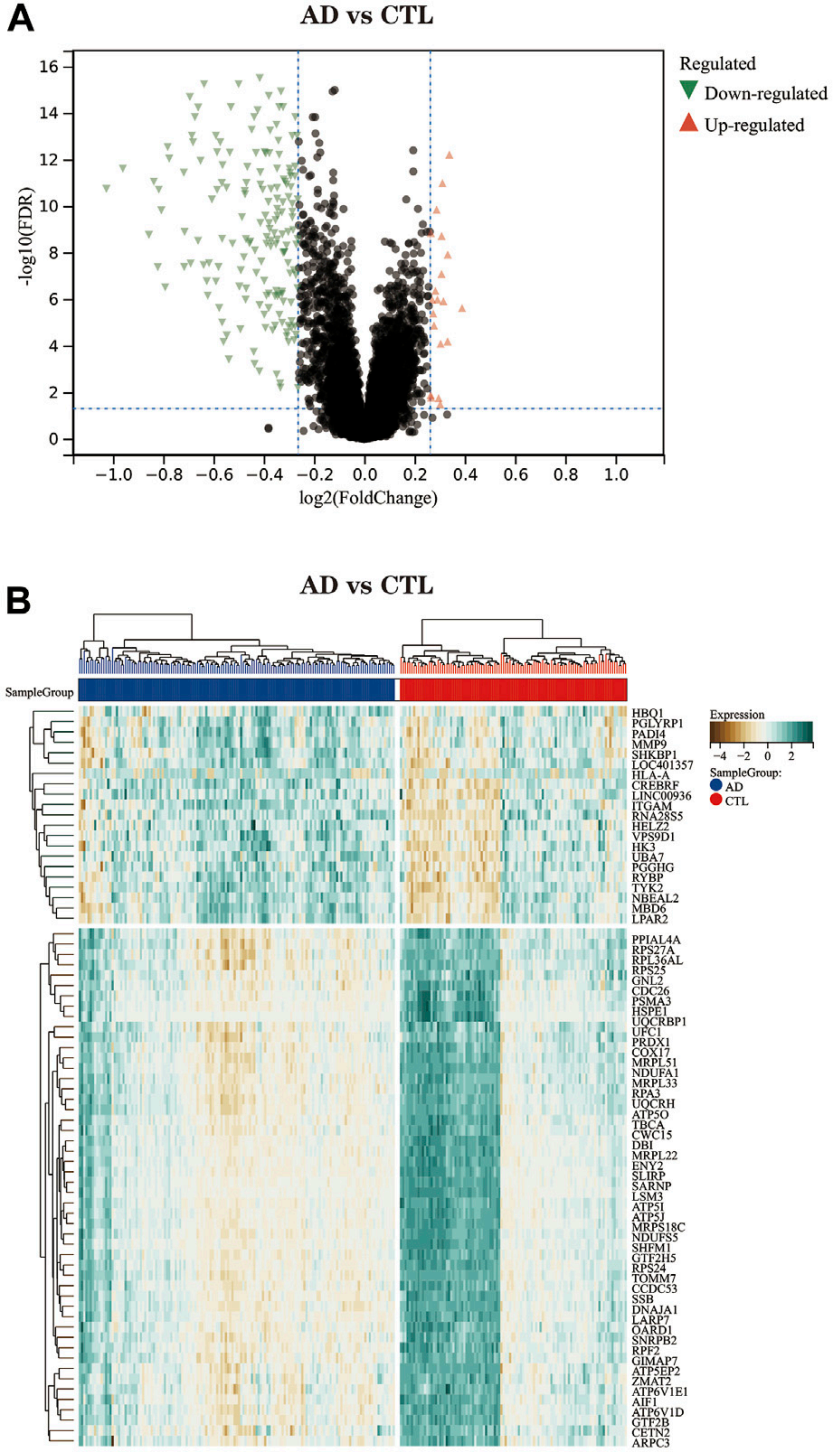
**(A)** The differential expression analysis results are shown in the volcano plot. Where the *x*-axis represents log2 (fold change) and the *y*-axis represents -log10 (adjust p. value). Green triangles represent downregulated genes, red triangles represent upregulated genes, and black dots represent genes with no obvious differential expression. **(B)** Heatmap of the top 50 differentially expressed genes. Each column in the graph represents a sample, each row represents a gene, and the expression status of the genes is indicated from high to low in brown to green, respectively, and at the top of the heat map, blue/red represents the AD group/CTL group, respectively.

The volcano plot (Figure 3A) shows that after differential expression analysis, we screened 200 differential expression genes (Supplementary Material S1), including 179 downregulated genes and 21 upregulated genes. The heat map (Figure 3B) shows the expression status of the top 50 differential expression genes.

### Construction of weighted gene co-expression network analysis and identification of core modules

Before performing the analysis, we first processed all pairs of genes using the Pearson correlation matrix and the average linkage method. Then, a weighted adjacency matrix is constructed, which is built by a power function, and we usually use the formula.

$$\mathrm{A mn}={\left|\mathrm{C mn}\right|}^{\beta }$$

**FIGURE 4:**
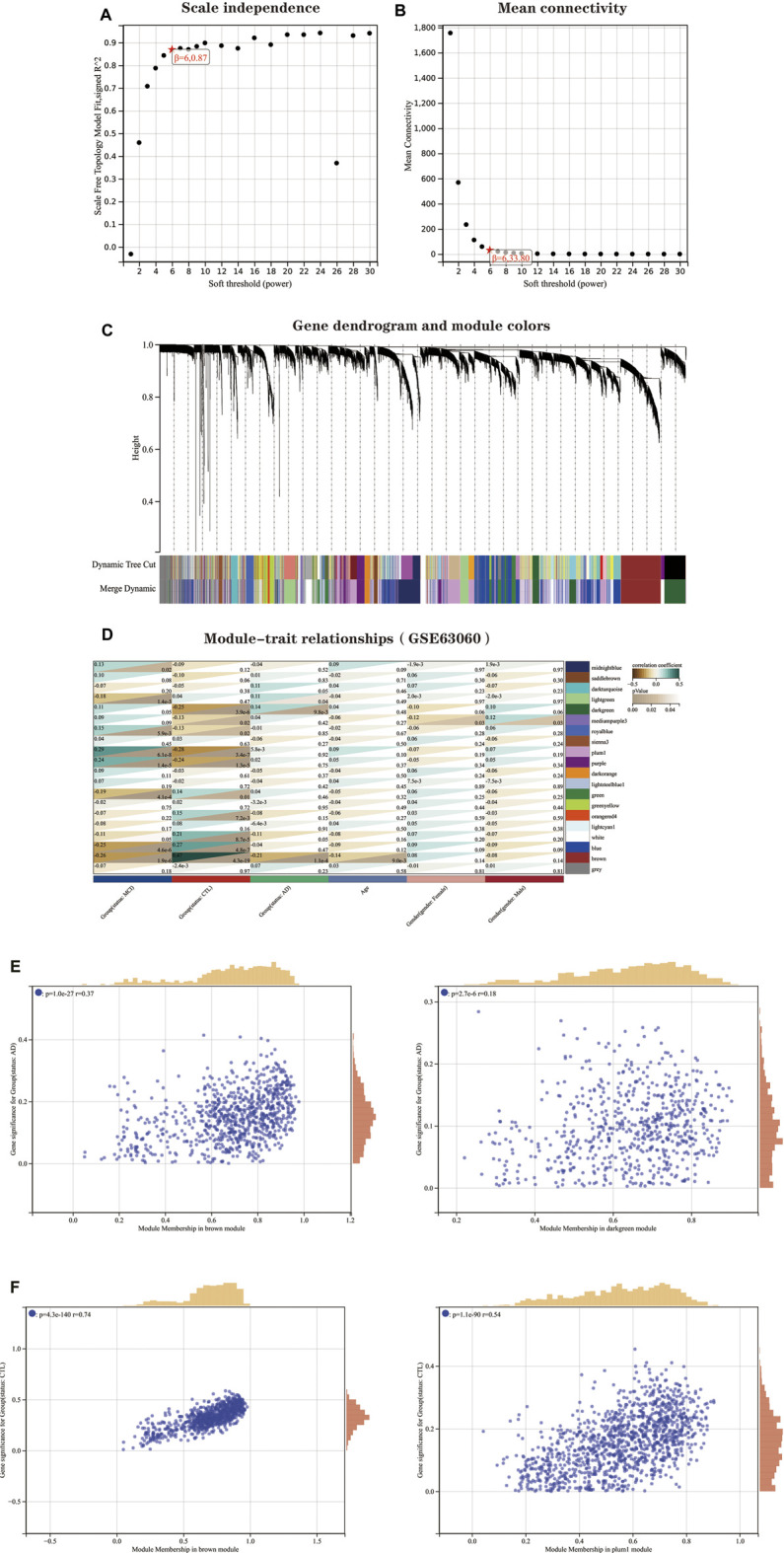
WGCNA of AD dataset GSE63060. **(A)** Scale-free index for analyzing the power of various soft thresholds. The horizontal coordinate represents the power of soft thresholds, and the best soft threshold is marked with an asterisk. **(B)** Average connectivity of various soft thresholds. **(C)** Identification of co-expressed gene modules. A dendrogram of all differentially expressed genes was clustered based on a measure of gene similarity. Cut lines of modules were identified, and a different color indicated each module. **(D)** Heat map of the correlation between modules and clinical phenotypes. Each row represents a module; each column represents a clinical trait. Each cell indicates the correlation between the module and the clinical phenotype. The corresponding cor value and *p*-value are labeled therein. The brown and dark green modules have the strongest correlation with AD, and the brown module and plum1 module have the strongest correlation with CTL.**(E)** Correlation between module membership (MM) and gene significance (GS) in the AD correlation module. r denotes the absolute correlation coefficient between GS and MM.**(F)**Correlation between module membership (MM) and gene significance (GS) in the CTL correlation module. r denotes the absolute correlation coefficient between GS and MM. Abbreviations: WGCNA (Weighted Gene Co-expression Network Analysis), AD (Alzheimer's Disease), CTL (Healthy Control).

Where C_mm is the Pearson correlation coefficient between gene m and gene n, and A_mn is the adjacency relationship between gene m and gene n. An important parameter in the construction of the weighted adjacency matrix is the soft threshold parameter β, which effectively emphasizes correlations between genes and, at the same time, penalizes weak correlations between genes. This study determines the soft threshold parameter as 6 (Figures 4A,B). Immediately after, we transformed this adjacency into a TOM matrix (topological overlap matrix), which better reflects the connectivity and adjacency between genes, and 1-TOM was defined as the difference between genes. To group genes with similar expression characteristics into the same module, we clustered genes in an average linkage hierarchy based on the dissimilarity measure of the TOM matrix. We set the minimum number of genes in the gene dendrogram to 30. We chose a cut line for the module dendrogram, calculated the similarity of module feature genes, and merged some similar modules to better delineate the modules (Figure 4C). After a series of calculations, we finally obtained 20 co-expression modules and visualized the correlation between modules and clinical features using the form of a heat map (Figure 4D). Notably, the grey module was considered a set of genes that could not be assigned to any module. From the correlation heat map of clinical phenotypes and modules, we could learn that the brown module negatively correlated with age and AD groups. In contrast, the genes in the mediumpurple3 module were differentially expressed between genders. The correlation between modules and clinical features was used to estimate the association of modules with features. Two methods were used to identify the key modules of the network. In the first method, Pearson correlation coefficients were calculated between the ME of each module and each clinical trait, allowing the identification of modules significantly associated with traits (p<0.05). In the second approach, the Pearson correlation coefficient [gene significance (GS)] between the expression level of each gene and each clinical trait was calculated; then, the mean absolute value GS of all genes in the module was calculated. The larger the mean total value, the stronger the correlation between the module and the clinical trait.

Also, we plotted the scatter plot of GS and MM correlations for each module (Figure 4E). Combining Figure 4D as well as Figures 4E,F, we can see that the brown module (cor = −0.21, *p* = 1.1e-4) and the dark green module (cor = 0.14, *p* = 9.8e-3) had the highest correlations with the AD group, and a total of 284 genes were extracted from these two modules (Supplementary Material S2). In contrast, the brown module (cor = 0.47, *p* = 4.3e-19) and the plum1 module (cor = −0.28, *p* = 3.4e-7) had the highest correlation with the CTL group, with a total of 335 genes extracted in the same way (Supplementary Material S3). The effect of clinical phenotype on module genes was also considered in the extraction of genes.

### Overlapping weighted co-expression networks-related module genes with differential expression genes

**FIGURE 5:**
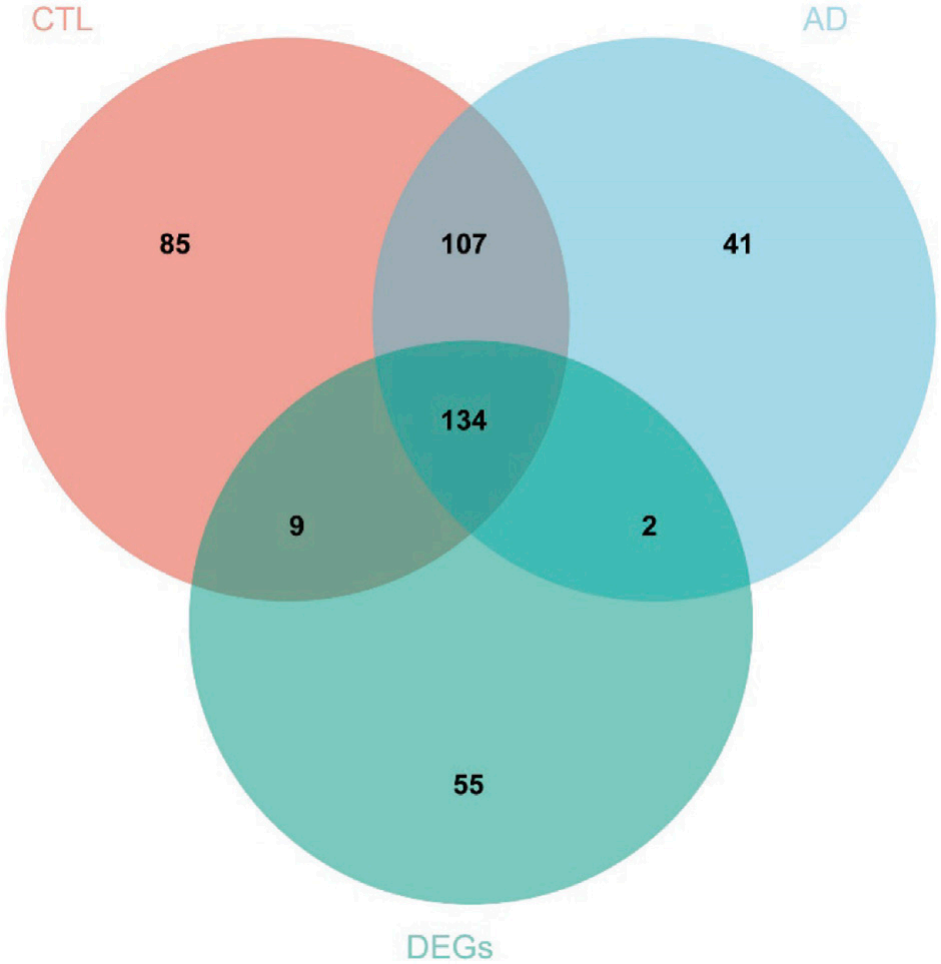
A total of 134 DEGs were screened for further analysis. Red represents 335 genes in the module with the strongest correlation to the CTL group, blue represents 284 genes in the module with the strongest correlation to the AD group, and the green represents 200 genes obtained from differential expression analysis. Abbreviations: AD (Alzheimer's Disease), CTL (Healthy Control), DEGs (Differential Expressed Genes).

We overlapped the genes derived from the genes obtained in the WGCNA analysis (335 genes in the CTL group-related module and 284 genes in the AD group-related module) and the 200 differentially expressed genes obtained in the differential expression analysis, and a total of 134 DEGs were screened for further analysis. The results were visualized by the Venn diagram (Figure 5) and recorded in the table (Supplementary Material S4).

### Kyoto encyclopedia of genes and genomes and gene ontology enrichment analysis for overlapping differential genes

**FIGURE 6:**
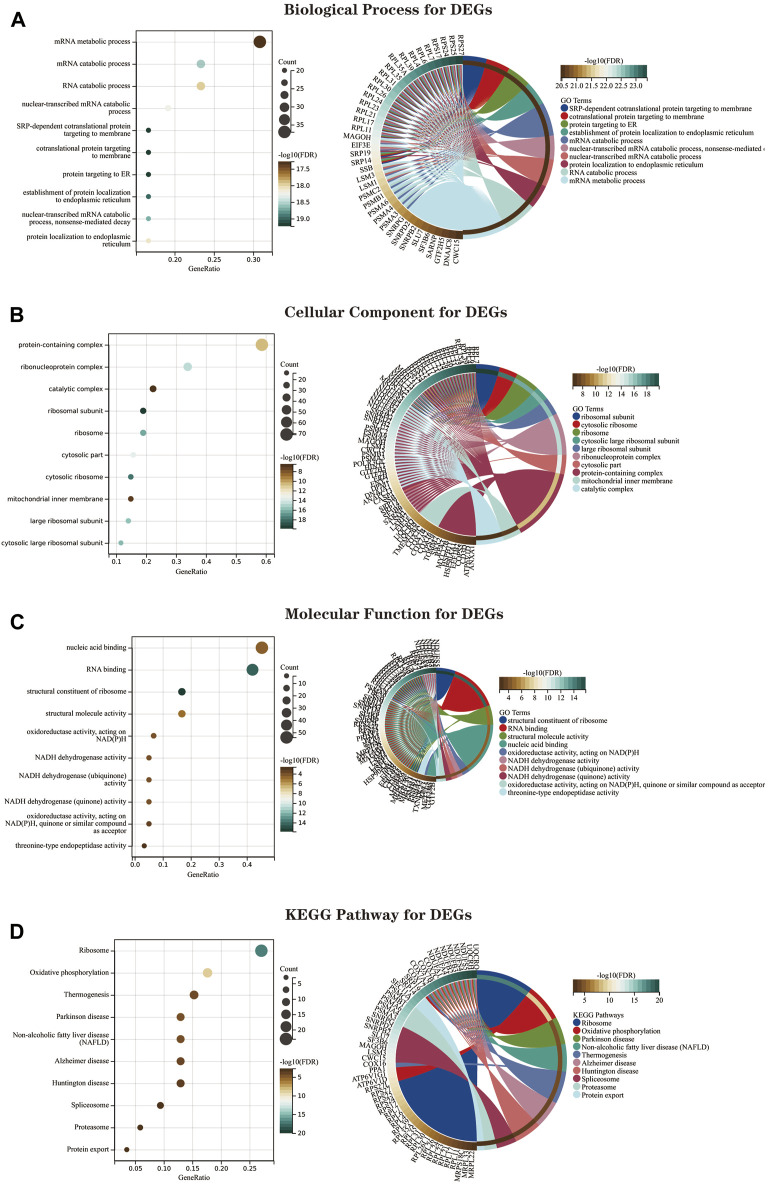
The GO and KEGG enrichment analysis results of 134 DEGs are shown as bubble and circle plots. **(A)** Shows the top 10 significantly enriched BP (biological process). **(B)** Shows the top 10 enriched CC (cellular component) considerably. **(C)** The top 10 enriched MF considerably (molecular function) are shown. **(D)** The top 10 significantly enriched KEGG pathways. In the bubble plot of GO and KEGG enrichment analysis, the *x*-axis represents the GeneRatio, the *y*-axis represents the -log10 (FDR) value, the bubble size represents the number of genes, and the color shades represent the size of the FDR value. The linkage between the left and right sides indicates the correlation between DEGs and terms. Abbreviations: DEGs (Differential Expressed Genes), GO (Gene Ontology), KEGG (Kyoto Encyclopedia of Genes and Genomes).

We performed GO and KEGG enrichment analysis on the screened 134 DEGs. The results of our GO enrichment analysis included BP (Figure 6A), CC (Figure 6B), and MF (Figure 6C). GO-BP was mainly enriched in RNA metabolic pathways such as mRNA metabolic process, mRNA catabolic process, and RNA catabolic process; GO-MF enrichment results showed DEGs were primarily associated with nucleic acid binding, RNA binding, structural constituent of ribosome, structural molecule activity, oxidoreductase activity, acting on NAD (P) H, and GO-CC analysis showed that these genes were significantly enriched in ribosomal structures such as protein-containing complex, ribonucleoprotein complex, catalytic complex, etc. The results of GO enrichment (Supplementary Material S5) indicate that DEGs play a role in ribosome function and mitochondrial function.

The KEGG pathway shows (Figure 6D; Supplementary Material S6)that DEGs are mainly involved in the “ribosome,” “oxidative phosphorylation,” “thermogenesis,” “Parkinson’s disease,” “NAFLD,” and “Alzheimer’s disease” pathways.

### Random forest screening for Alzheimer’s disease hub genes

**FIGURE 7:**
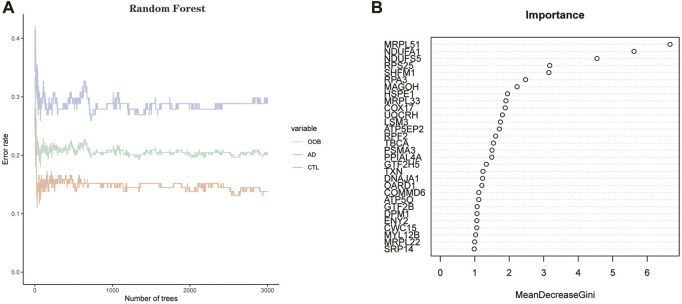
**(A)** Plot of decision tree versus error. The *x*-axis represents the number of decision trees; the *y*-axis represents the error. **(B)** Screening of hub genes by Gini coefficient method. The *X*-axis represents the importance index, the *y*-axis represents the DEGs, and all DEGs are ranked according to the “mean reduction Gini coefficient.” The higher the value, the closer the relationship between the gene and the disease.

We imported the expression profile files of 134 differentially expressed genes into the random forest model. Before calculation, firstly, we set the random seed to 123,456 and calculate the number of decision trees needed to achieve the highest accuracy of the model in cross-validation. The optimal number of decision trees is 72 by operation. Next, we construct the random forest model. Regarding parameter settings, “importance” is the parameter for judging the importance of variables, the “proximity” parameter is used to set the proximity matrix for calculating the model, and “ntree” is used to set the number of random forest decision trees. The importance score of each gene in the random forest model was calculated using the Gini coefficient method. Specifically, it is a method of decreasing accuracy. The relationship between the random forest model error and the number of decision trees is shown in the following figures (Figures 7A,B). The genes with a better importance score than two were selected as the hub genes for the subsequent analysis. MRPL51, NDUFA1, NDUFS5, RPS25, SHFM1, RPA3, and MAGOH, respectively. Among these genes, NDUFS5, SHFM1, RPA3, and MAGOH have never been mentioned or confirmed associated with AD development in studies.

**FIGURE 8:**
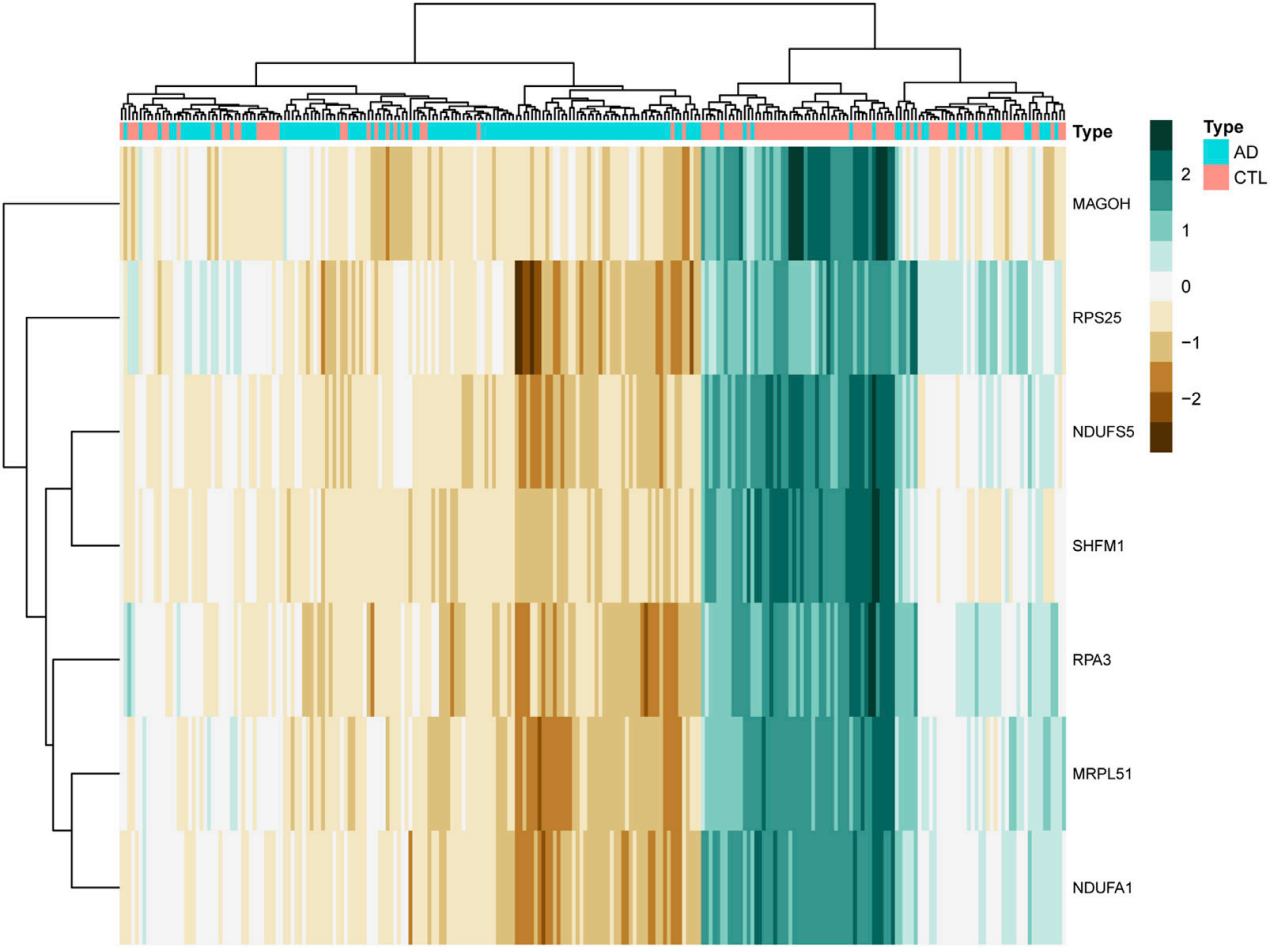
The clustering heat map shows the clustering results of the seven hub genes screened by the random forest algorithm in the GSE63060 dataset. The brown color represents the highly expressed genes in the samples, the green color represents the lowly expressed genes in the samples, the blue color at the top of the heat map represents the AD group samples, and the red color represents the CTL group samples.

We clustered the GSE63061 dataset and plotted a heat map (Figure 8), confirming that the seven hub genes mentioned above perform well in distinguishing between diseased and normal samples.

### Correlation analysis between hub genes and immune characteristics

**FIGURE 9:**
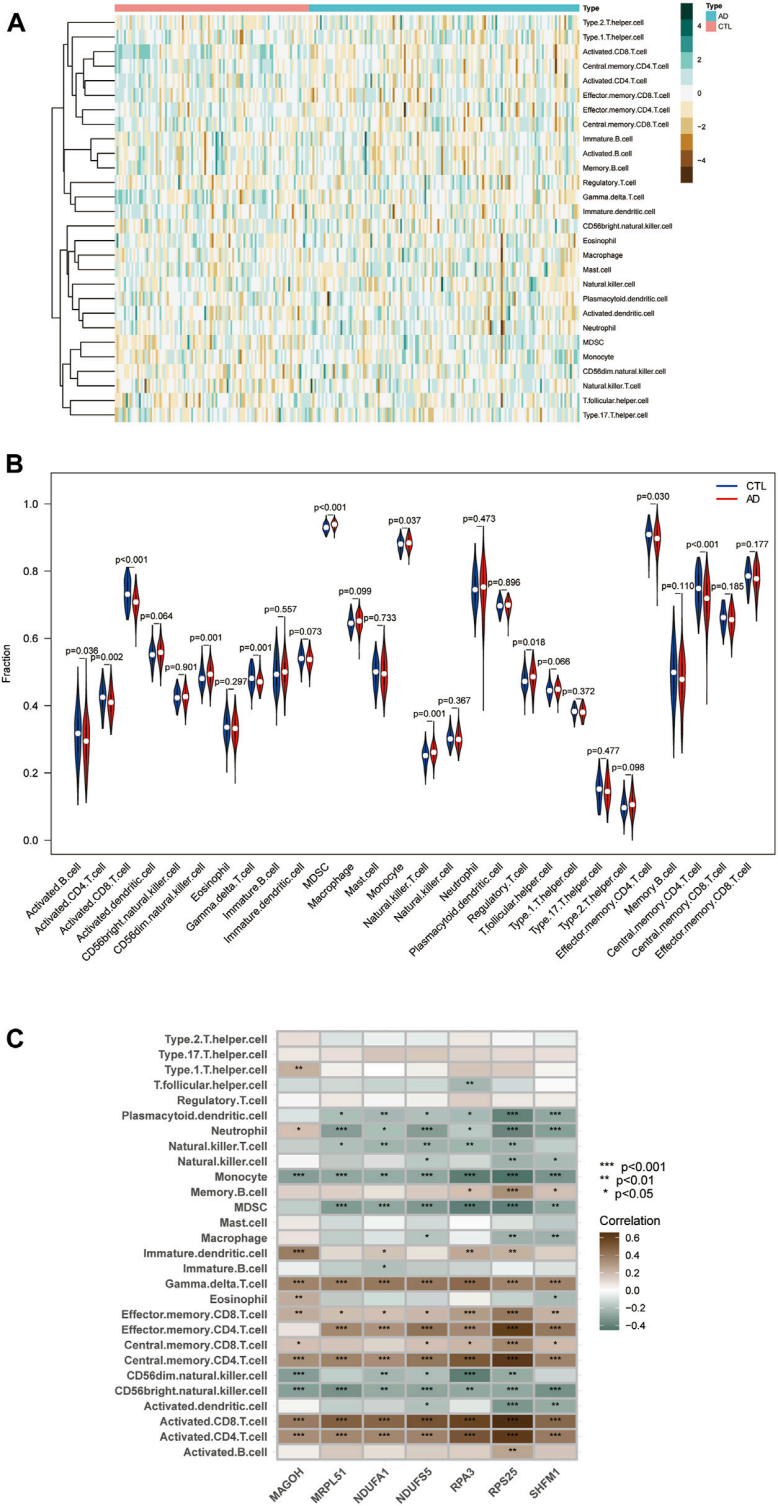
Immune infiltration landscape between AD and CTL obtained by ssGSEA analysis. **(A)** Heat map summarizing the scores of immune cell infiltration between AD patients and non-AD patients. **(B)** Violin plot showing the difference in immune cell infiltration between AD (red) and CTL (blue), *p* < 0.05, was considered statistically significant. **(C)** Shows the correlation between hub genes and immune cells. The colors from brown to green represent the change from positive to negative correlations, respectively. More asterisks and darker colors of the modules represent stronger correlations.

We quantified the immune infiltration scores of 28 immune cells in the samples using the method of ssGSEA. We plotted heat maps showing the distribution of immune cells in different samples and the infiltration scores (Figure 9A; Supplementary Material S7). The discrepancy in immune cell infiltration between the AD and CTL groups was then computed and visualized the results. The results showed (Figure 9B; Supplementary Material S8) that the proportion of CD56dim.natural.killer. Cells, MDSC, Monocyte, Natural. killer.T.cells, and Regulatory.T.cells were substantially higher in the AD group than in the CTL group. And many cells had lower fractions than normal patients, such as Activated.B.cells, Activated. CD4.T.cells, Activated. CD8.T.cells, Gamma. delta.T.cells, Effector. memory.CD4.T.cells, Central. CD4.T.cell.

We analyzed the correlation between immune cell infiltration scores and hub genes using Spearman correlation to explore the role played by hub genes in the immune microenvironment and the corresponding mechanisms. The results are shown (Figure 9C): the seven hub genes identified by the random forest algorithm were strongly correlated with the level of immune cell infiltration, suggesting that these genes may play a role in the development of AD by regulating the immune microenvironment.

### Construction and validation of artificial neural network models

First, the expression profile data of the seven hub genes identified by the random forest algorithm were imported. Normalization of the input data was used to normalize the data. The input variables were normalized through the input nodes, and the normalized values fell between 0 and 1, or −1 to 1. We chose min-max (0,1) and performed the extrapolation. When choosing the parameters, we set the number of hidden layers to 5. There is no fixed rule for the number of hidden layers and the number of input neurons. The number of neurons is generally between two-thirds of the input layer size and one-third of the output layer size. In this study, the number of neurons is set to 7. The training and validation sets used to train the model are created randomly from the input data set. The purpose of the training set is to calculate the importance value score (gene weights) for each candidate gene. And the validation set is used to test the classification performance of the model scores using the expression of genes and gene weights. Finally, the formula was used.

neural AD = Gene Expression× Gene Weight

**FIGURE 10:**
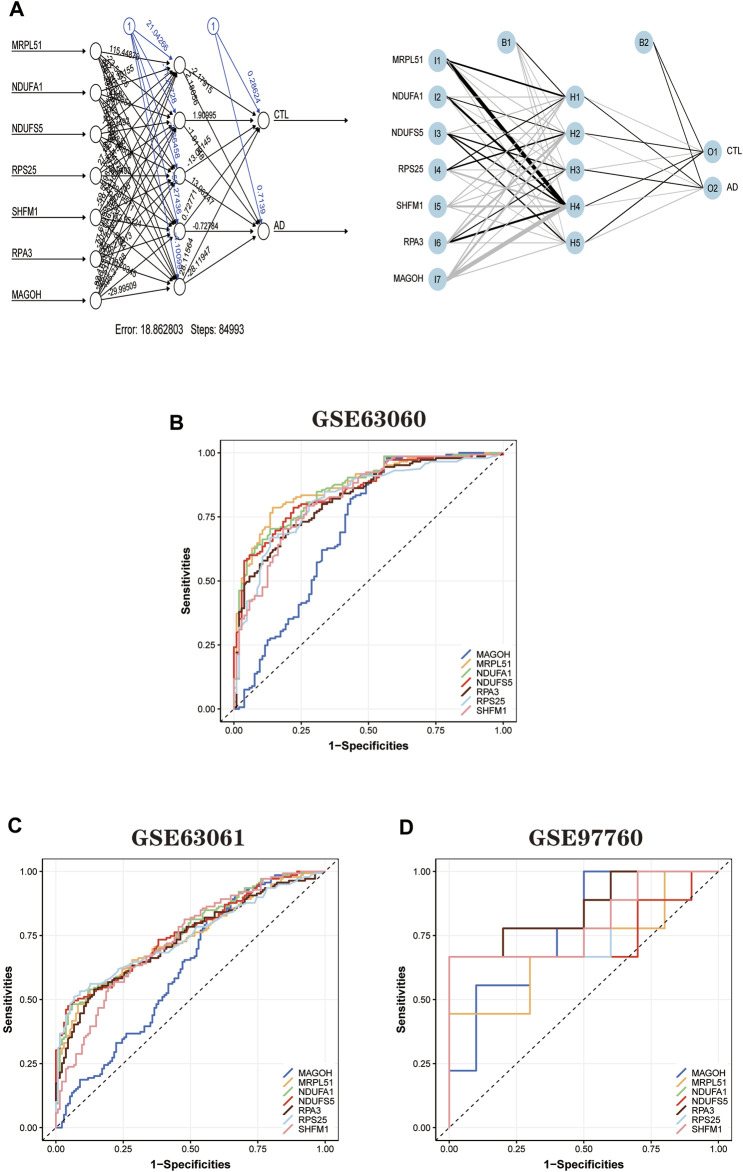
Artificial neural network model building and ROC curve validation. **(A)** Visualization of the artificial neural network model has undergone 84,993 training sessions and contains five hidden layers and two output layers. **(B)** ROC curves of the training set GSE63060 dataset. **(C)** The validation results of ROC curves in the validation set GSE63061 dataset. **(D)** The validation results of ROC curves in the validation set GSE97760 dataset. The different color lines represent different genes.

The disease neural network classification score neural AD is obtained. The specific training process is as follows: ① The initial value of the network weights is set to 0, and the function of each node estimates the target variable value of the data. ② Compare the error between the actual and estimated values and readjust the bias of each weight according to the error value. Step ① is repeatedly executed until the error between the actual and calculated values is minimized, at which point learning is stopped to obtain the best weights. The model’s training process went through a total of 84,993 steps, and the termination condition (reaching the threshold) was the absolute partial derivative of the error function < 0.01. The output results of the artificial neural network model and the weight information of the candidate genes are shown in the table (Figure 10A; Supplementary Material S9). The accuracy of the artificial neural network model is reflected by the AUC values of the hub genes, and the larger the value, the higher the accuracy of the model is proved. We calculated the AUC values of the hub genes (Figure 10B): MRPL51 (0.87), NDUFA1 (0.86), NDUFS5 (0.85), RPS25 (0.82), SHFM1 (0.83) RPA3 (0.83), and MAGOH (0.81).

In addition, to further validate the accuracy of the ANN model, two independent datasets (GSE63061 and GSE97760) were selected for analysis. During the validation of the ANN model accuracy using the independent dataset GSE63061, we calculated the AUC values of seven hub genes using the same method (Figure 10C): MRPL51 (0.74), NDUFA1 (0.76), NDUFS5 (0.76), RPS25 (0.74), SHFM1 (0.73), RPA3 (0.73), and MAGOH (0.62). It is worth noting that the AUC values of the above genes remained significant when validated using the independent dataset GSE97760 (Figure 10D) for MRPL51 (0.69), NDUFA1 (0.77), NDUFS5 (0.74), RPS25 (0.79), SHFM1 (0.80), and RPA3 (0.86), and MAGOH (0.78). The validation results confirm that the ANN model has good classification performance for AD and normal samples.

## Discussion

In this study, we used a combination of bioinformatics analysis and machine learning to obtain differential genes (DEGs) for AD serology and did GO and KEGG enrichment analysis. We got the following results from the enrichment analysis of the obtained DEGs. GO analysis showed that DEGs were significantly enriched in ribosomal and mitochondrial functions. The KEGG pathway leads that DEGs are mainly involved in “ribosome,” “oxidative phosphorylation,” “thermogenesis,” “Parkinson’s disease,” and “non-alcoholic fatty liver disease (NAFLD)” and “Alzheimer’s disease” pathways. Previous studies have confirmed the role of the ribosomal (Ding et al., 2005; Nyhus et al., 2019) and oxidative stress pathways (Simunkova et al., 2019; Butterfield and Mattson, 2020; Zhang et al., 2020) in AD development, which deserves further exploration.

Then seven hub genes MRPL51, NDUFA1, NDUFS5, RPS25, SHFM1, RPA3, and MAGOH were obtained by a random forest algorithm. Among these seven hub genes, NDUFA1 and MRPL51 were considered potential biomarkers of AD in previous bioinformatics analyses (Li et al., 2018; Liu et al., 2021). NDUFA1 is an essential component of the human respiratory chain complex I. It is involved in mitochondrial function and oxidative phosphorylation and is a critical coding gene in the human body. Some studies have confirmed that partial deletion of respiratory chain function may impair ATP synthesis and chronic increase of oxidative stress (Carelli et al., 2002). A survey of optic neuropathy also suggested that reduced gene expression of mitochondrial proteins leads to neuronal degeneration (Qi et al., 2003). Both studies indicate that downregulation of the NDUFA1 gene in the serum of AD patients is likely to lead to impaired oxidative phosphorylation and partial deficiency of mitochondrial function, which is ultimately involved in the disease development of AD. In other studies, it has been suggested that RPS25 and AIF1 may also play a role in AD development (Sanfilippo et al., 2020; Wang et al., 2021). In contrast, one study confirmed that RPS25 is a therapeutic target for neurodegenerative diseases caused by nucleotide repeat amplification, which indirectly confirms the role of RPS25 in the pathophysiological process of AD (Yamada et al., 2019). However, few studies have mentioned or established the association of NDUFS5, SHFM1, RPA3, and MAGOH genes with AD development. NDUFS5 is a member of the iron-sulfur family of NADH dehydrogenases (ubiquinone) and encodes a subunit of the mitochondrial respiratory chain complex I (Wirth et al., 2016). Previous studies have highlighted the role of mitochondrial dysfunction in AD (Cai and Tammineni, 2017; Perez Ortiz and Swerdlow, 2019), leading us to speculate that NDUFS5 may be involved in the pathogenesis of AD by affecting mitochondrial function and oxidative phosphorylation processes. SHFM1 encodes the 26S proteasome subunit, one of the proteasome components. Earlier studies have confirmed the role of the proteasome in inhibiting neurodegeneration and that impaired proteasome function occurs in the early stages of AD (Keller et al., 2000; Cecarini et al., 2007). The differential expression of SHFM1 in AD patients is likely to be a manifestation of proteasome dysfunction, offering the possibility of its use as a biomarker for early screening of AD. RPA3 is a protein-coding gene mainly involved in DNA repair and DNA replication. It has been shown that disruption of DNA repair may lead to increased DNA damage in AD patients and increase the risk of AD, providing a theoretical basis for RPA3 as a biomarker for AD. MAGOH is a protein-coding gene involved in the development of the nervous system. Little research has been done on this gene, and further studies are needed to elucidate its potential association with AD.

We further constructed a diagnostic model for AD using artificial neural networks based on the above seven hub genes. We validated the efficacy of the model in two publicly available datasets. The bioinformatics analysis combined with the machine learning approach is the innovation of this study, and good results were obtained. The random forest (RF) algorithm is an emerging and high precision machine learning algorithm that has been widely used in numerous fields, and of course, its role in the medical field is also exact. RF algorithms have been used for clinical diseases, such as using random forests to identify biomarkers for glioblastoma to find potential targets for treatment (Li et al., 2021), building COPD risk prediction models (Perret et al., 2021), and detecting and predicting type 2 diabetes (Muneeb and Henschel, 2021), all with good results. An artificial neural network is a new type of algorithm derived from imitating the structure and function of the human brain, which has the characteristics of self-learning ability and high efficiency compared with the traditional machine learning algorithm. It has also found many applications in clinical settings. Studies have been using artificial neural network models to accurately predict the risk of liver failure after hepatectomy in patients with hepatocellular carcinoma who underwent hemihepatectomy (Mai et al., 2020). Artificial neural networks have also been used in AD for a long time. Some scholars have applied artificial neural networks to the diagnosis of AD based on the information contained in the digital images of SPECT cerebral blood flow assessment (Świetlik and Białowąs, 2019). There is a precedent for combining two machine learning algorithms to diagnose and predict diseases (Mozafari et al., 2020; Xie et al., 2020). Still, it is noteworthy that no research has yet used this combination of the two in the field of AD (Feng et al., 2021). Therefore, combining random forests and artificial neural networks to build AD diagnosis models is a bold attempt and an excellent complement to the existing diagnosis methods. At the same time, our study revealed AD susceptibility genes that may be involved in the regulation of mitochondrial function and ribosomal pathways. We hope that their essential value will be reflected in future studies. Meanwhile, immunoassays showed that hub genes are closely related to immune cell infiltration, confirming that dysregulation of the immune microenvironment plays an essential role in the pathogenesis of AD.

However, there are limitations to this study. First, AD is highly heterogeneous, which affects our understanding and judgment of the disease (Jellinger, 1996). Second, some AD patients have other neurodegenerative lesions in combination, affecting the model’s accuracy. And the data sample used in this study is still insufficient, and the sample size needs to be increased and further studied and optimized. Biological experiments for critical steps may be more revealing, but they cannot be completed at this time for objective reasons. In subsequent studies, we will continue to analyze these genes to further their upstream and downstream pathways to understand AD’s biomolecular mechanisms better.

## Conclusion

Using bioinformatics analysis and machine learning algorithm modeling, we uncovered potential biomarkers of AD based on immune cell infiltration while constructing a random forest and artificial neural network AD diagnostic model. We confirmed its excellent classification performance in two independent datasets. This study nicely complements the existing tools for early screening and diagnosis of AD and reveals AD susceptibility genes that may be involved in the regulation of mitochondrial function and ribosomal function; and also provides new perspectives for a better understanding of molecular immune mechanisms and finding drug targets.

## Data Availability

Publicly available datasets were analyzed in this study. The names of the repository/repositories and accession number(s) can be found in the article/Supplementary Material
